# Supplementary material for: Finding the best trade-off between performance and interpretability in predicting hospital length of stay using structured and unstructured data
Source: PLoS One. 2023 Nov 30;18(11):e0289795. doi: 10.1371/journal.pone.0289795 (PMC10688642; doi:10.1371/journal.pone.0289795)

# Appendix 1 – Confusion Matrices of Baseline Random Models

Baseline values assuming a priori distribution predicted values

|  |  | **Predicted** | |  |
| --- | --- | --- | --- | --- |
|  |  | 0 | 1 | Total |
| **Actual** | 0 | 26448.95 | 2076.05 | 28525 |
|  | 1 | 2076.05 | 162.95 | 2239 |
|  | Total | 28525 | 2239 | 30764 |
|  |  |  |  |  |
|  | **Accuracy** | 0.865 |  |  |
|  | **Precision** | 0.073 |  |  |
|  | **Recall** | 0.073 |  |  |
|  |  |  |  |  |
|  | **F1 =** | 0.073 |  |  |

Baseline values assuming a uniform distribution predicted values

|  |  | **Predicted** | |  |
| --- | --- | --- | --- | --- |
|  |  | 0 | 1 | Total |
| **Actual** | 0 | 14262.5 | 14262.5 | 28525 |
|  | 1 | 1119.5 | 1119.5 | 2239 |
|  | Total | 15382 | 15382 | 30764 |
|  |  |  |  |  |
|  | **Accuracy** | 0.5 |  |  |
|  | **Precision** | 0.074 |  |  |
|  | **Recall** | 0.500 |  |  |
|  |  |  |  |  |
|  | **F1 =** | 0.127 |  |  |

An example of baseline values for a balanced dataset

|  |  | Predicted | |  |
| --- | --- | --- | --- | --- |
|  |  | 0 | 1 |  |
| Actual | 0 | 7691 | 7691 | 15382 |
|  | 1 | 7691 | 7691 | 15382 |
|  |  | 15382 | 15382 | 30764 |
|  |  |  |  |  |
|  | Accuracy | 0.500 |  |  |
|  | Precision | 0.500 |  |  |
|  | Recall | 0.500 |  |  |
|  |  |  |  |  |
|  | F1 = | 0.500 |  |  |

# Appendix 2 – Topics Description

Table A2.i Topics description, Stemming

| **TF** | | | | | |  |
| --- | --- | --- | --- | --- | --- | --- |
| **Topic F274** | **Topic F87** | **Topic F195** | **Topic F32** | **Topic F242** | **Topic F92** | |
| tube | collect | wound | liver | topic | extrem | |
| feed | fluid | foot | transplant | appl | lower | |
| tube feed | drain | dress | feed | one appl | lower extrem | |
| place | fluid collect | debrid | tube | appl topic | leg | |
| tracheostomi | abscess | vac | liver transplant | sig | left lower | |
| wean | drainag | tissu | start | sig one | upper | |
| requir | ct | plastic | hepat | miconazol | upper extrem | |
| start | place | skin | tube feed | time day | right lower | |
| ventil | abdomin | chang | increas | nitrat | left leg | |
| respiratori | tpn | graft | level | miconazol nitrat | bilater lower | |
| cultur | start | left foot | done | cream | right leg | |
| status | increas | flap | note | powder | swell | |
| nutrit | cathet | plastic surgeri | feed tube | nitrat powder | skin | |
| also | iv | open | alk phos | topic time | surgeri | |
| toler | leak | closur | requir | day need | deep | |
| vancomycin | chang | wound vac | sicu | powder sig | tibial | |
| remain | site | skin graft | phos | cream sig | right upper | |
| placement | tube | vac dress | negat | tablet sig | calf | |
| per | decreas | surgeri | alk | daili daili | vein | |
| support | output | soft tissu | decreas | one tablet | poplit | |

| **TF** | | **BIN** | | | |  |
| --- | --- | --- | --- | --- | --- | --- |
| **Topic F219** | **Topic F294** | **Topic F99** | **Topic F43** | **Topic F27** | **Topic F255** | |
| postop | extrem | antibiot | wound | postop | liver | |
| postop day | show | cultur | dress | oper | hepat | |
| oper | neurolog | vancomycin | debrid | diet | ascit | |
| remain | command | infecti | dress chang | exploratori | portal | |
| unit | pupil | infecti diseas | vac | laparotomi | dilat | |
| surgeri | head | line | closur | toler | us | |
| toler | follow command | nutrit | tissu | postop day | biliari | |
| room | ct | scan | open | exploratori laparotomi | vein | |
| post | move | support | drainag | abdomin | portal vein | |
| diet | awak | develop | surgeri | surgeri | distend | |
| oper room | reactiv | blood cultur | collect | advanc | abdomin | |
| note | head ct | tube | heal | post | ultrasound | |
| place | upper | broad | fluid collect | drain | abdomin pain | |
| status | remain | spike | incis | regular diet | gallbladd | |
| regular | alert | ct scan | site | place | amount | |
| without | eye | requir | surgeri allergi | regular | lesion | |
| underw | pressur | persist | common | taken | ruq | |
| remov | equal | feed | servic surgeri | nausea | pancreat | |
| taken | open | empir | femor | oper room | cirrhosi | |
| status post | drain | coverag | superfici | underw | patent | |

| **BIN** | | | | | **TF-IDF** |
| --- | --- | --- | --- | --- | --- |
| **Topic F286** | **Topic F210** | **Topic F12** | **Topic F141** | **Topic F202** | **Topic F1** |
| intraven | command | medicin allergi | sensat | puls femor | mg po |
| soln | follow command | servic medicin | nerv | full rom | qd |
| recon soln | head ct | medicin | reflex | radial | po qd |
| recon | head | ed | strength | rom | dictat |
| soln sig | neurolog | fever | symmetr | suppl full | status post |
| chewabl | move | cxr | cranial | chest lung | status |
| chewabl po | ct | infect | light | perrla eomi | unit |
| tablet chewabl | hemorrhag | cultur | cranial nerv | perrla | hematocrit |
| carbon | open | given | sensat intact | nondistend nontend | pressur |
| calcium carbon | intub | symptom | tongu | dp | examin |
| chewabl sig | eye | heent | intact | soft nondistend | post |
| picc | show | bp | touch | dri intact | blood pressur |
| vancomycin | open eye | negat | motor | clear bilater | care unit |
| calcium | neurosurgeri | like | toe | wellperfus | rate |
| 500 mg | extrem | deni | light touch | warm wellperfus | medquist36 |
| vitamin | repeat | initi | facial | skin dri | dictat medquist36 |
| acid | scan | chill | face | femor | job |
| 500 | 5000 | diarrhea | drift | extrem warm | show |
| disposit extend | upper extrem | primari | neurolog | heart rrr | intens care |
| care facil | sedat | improv | orient | nontend bowel | also |

| **TF-IDF** | | | | |
| --- | --- | --- | --- | --- |
| **Topic F123** | **Topic F162** | **Topic F57** | **Topic F60** | **Topic F21** |
| marrow | sig | aortic | ct | repeat head |
| bone marrow | sig one | sig | fluid | drain |
| lymphoma | tablet sig | valv | mass | command |
| marrow biopsi | one tablet | sig one | note | head ct |
| bone | pt | refills0 | bowel | follow command |
| biopsi | capsul | tablet sig | within | upper extrem |
| leukemia | po daili | releas | new | open eye |
| chemotherapi | everi | one tablet | pleas | extrem |
| neutropenia | time day | mitral | lesion | move |
| 1200am | daili daili | releas ec | vomit | head |
| 1200am blood | sp | aortic valv | cancer | ct show |
| lymphocyt | pleas | delay releas | metastat | awak |
| acyclovir | plt | ec | small | pupil |
| cell | releas | tablet delay | abdomin | open |
| urean7 | ml | disp30 | lobe | awak alert |
| omr | everi hour | tablet refills0 | pancreat | move extrem |
| parenchyma | po bid | disp30 tablet | like | neurolog stabl |
| patient admit | bid | delay | tube | repeat |
| parenchym | delay | coronari | chang | eye |
| patient acut | take | capsul | evid | tomographi |

Table A2.ii Topics description, Lemmatization

| **TF** | | | | | |  |
| --- | --- | --- | --- | --- | --- | --- |
| **Topic F268** | **Topic F240** | **Topic F180** | **Topic F88** | **Topic F60** | **Topic F174** | |
| tube | intubate | abscess | transplant | tpn | left | |
| feed | respiratory | collection | liver | fluid | leave | |
| place | intubation | fluid collection | liver transplant | collection | leg | |
| tube feed | extubate | fluid | increase | pancreatic | extremity | |
| wound | respiratory failure | ct | bid | ct | low extremity | |
| placement | sedate | drainage | level | fluid collection | fracture | |
| surgery | tube | drain | note | start | low | |
| require | failure | scrotal | drain | necrotize | hip | |
| nutrition | extubation | left | prograf | drain | femur | |
| note | osh | scan | lab | sig | thigh | |
| tolerate | endotracheal | ct scan | cirrhosis | change | ankle | |
| remain | pneumonia | infection | sicu | pseudocyst | knee | |
| tracheostomy | give | sig | output | drainage | wound | |
| start | likely | show | demonstrate | place | surgery | |
| drain | start | infectious | start | new | orthopedic | |
| peg | patient intubate | place | po bid | pancreatitis | distal | |
| drainage | sedation | large | prednisone | abd | left leg | |
| also | intubate sedate | likely | fluconazole | necrosis | orif | |
| open | fio2 | cm | fluid | pancrea | joint | |
| perform | cxr | fever | tacrolimus | abdomen | skin | |

| **TF** | | | | | **BIN** |
| --- | --- | --- | --- | --- | --- |
| **Topic F16** | **Topic F101** | **Topic F87** | **Topic F86** | **Topic F251** | **Topic F258** |
| wound | vascular | extremity | tube | appl | tube |
| cellulitis | foot | command | feed | topical | feed |
| ulcer | disease | show | tube feed | one appl | place |
| change | peripheral | head | respiratory | appl topical | tube feed |
| debridement | vascular disease | follow command | status | sig | surgery |
| foot | peripheral vascular | ct | prn | sig one | operating |
| tissue | toe | eye | pneumonia | miconazole | operating room |
| infection | vascular surgery | pupil | pressure | miconazole nitrate | unit |
| dress | surgery | move | wean | nitrate | room |
| vac | bypass | left | support | topical time | scan |
| osteomyelitis | left | head ct | per | time day | tolerate |
| skin | leave | remain | place | cream | antibiotic |
| wound care | amputation | upper | unit | nitrate powder | status |
| dressing | pulse | open | ventilator | lotion | also |
| erythema | ulcer | pressure | tracheostomy | day need | culture |
| leg | right foot | low extremity | percutaneous | cream sig | placement |
| dress change | leg | drain | start | topical qid | show |
| low | pvd | repeat | vancomycin | 505 | intubate |
| sig | femoral | awake | sputum | camphormenthol | post |
| soft tissue | tibial | upper extremity | require | powder sig | intensive |

| **BIN** | | | | | |  |
| --- | --- | --- | --- | --- | --- | --- |
| **Topic F27** | **Topic F184** | **Topic F81** | **Topic F232** | **Topic F163** | **Topic F284** | |
| biopsy | lactulose | dialysis | appl | mg5 ml | appl | |
| lesion | cirrhosis | meal | miconazole | mg5 | one appl | |
| cancer | lactulose 10 | day meal | powder | ml | topical | |
| cell | ascite | hemodialysis | topical | mls | appl topical | |
| metastatic | portal | renal | one appl | ml po | miconazole nitrate | |
| mri | varix | insulin | nitrate | solution | miconazole | |
| diagnose | liver | calcium | miconazole nitrate | suspension | nitrate | |
| chemotherapy | ml syrup | wmeal | nitrate powder | solution sig | topical time | |
| mass | syrup | tid wmeal | appl topical | mls po | nitrate powder | |
| bone | gram15 | unitml | unitml | every hour | cream | |
| oncology | 10 gram15 | hd | trach | suspension sig | cream sig | |
| ct | gram15 ml | acid | unitml solution | hour need | powder sig | |
| nodule | hepatic | po tid | picc | ml solution | lotion | |
| metastasis | 30 ml | esrd | tracheostomy | q6h every | topical qid | |
| consistent | syrup sig | wmeal time | peg | disposition extended | day need | |
| tumor | ml po | slide | porcine | mg suppository | qid time | |
| disease | spironolactone | slide scale | solution | extended care | topical tid | |
| treatment | liver disease | scale | injection | suppository | 505 | |
| malignancy | thirty 30 | 13 | 17 | q6h | camphormenthol | |
| scan | thirty | acid mg | heparin | extended | 0505 lotion | |

| **BIN** | | | **TF-IDF** | | |  |
| --- | --- | --- | --- | --- | --- | --- |
| **Topic F80** | **Topic F279** | **Topic F277** | **Topic F59** | **Topic F205** | **Topic F198** | |
| orthopedic | transplant | aneurysm | small bowel | mg po | aortic | |
| fracture | mycophenolate | headache | ostomy | examination | valve | |
| sodium 100 | mofetil | strength | drain | status | question concern | |
| every hour | mycophenolate mofetil | head | collection | hematocrit | incision | |
| docusate sodium | prednisone | hemorrhage | abdominal | status post | sig | |
| surgery | tacrolimus | symmetric | tpn | dictate | please | |
| docusate | cellcept | drift | laparotomy | care unit | artery | |
| leg | service surgery | tongue | fluid | unit | leave | |
| allergie | chill nausea | visual | abscess | medquist36 | coronary | |
| hour need | chill | cerebral | exploratory | dictate medquist36 | cardiac surgery | |
| every | 450 | neurosurgery | exploratory laparotomy | job | surgery office | |
| capsule sig | liver | awake | bowel | blood pressure | coronary artery | |
| tablet sig | sicu | head ct | fluid collection | intensive care | surgeon | |
| sig | lab | midline | ileostomy | intensive | call cardiac | |
| sodium | datetime | angiogram | wound | show | please call | |
| sig one | fluconazole | cranial | small | blood cell | answer service | |
| capsule po | nausea | intact | obstruction | rate | concern answer | |
| allergie patient | nausea vomit | nerve | tube | post | person hour | |
| drug attend | monday | artery | pancreatitis | pressure | call person | |
| allergy drug | surgery allergie | fluent | pancreatic | count | contact call | |

| **TF-IDF** | | | |
| --- | --- | --- | --- |
| **Topic F122** | **Topic F123** | **Topic F233** | **Topic F121** |
| cirrhosis | ml | sig | aortic |
| varix | trach | sig one | refills0 |
| liver | recon | urine | valve |
| lactulose | line | give | disp30 |
| ascite | unitml | pt | coronary |
| portal | recon soln | pneumonia | disp30 tablet |
| egd | flush | one tablet | dr week |
| nadolol | insulin | tablet sig | tablet refills0 |
| hepatic | soln | failure | mitral |
| paracentesis | picc | mg po | daily disp30 |
| encephalopathy | solution | renal | release |
| esophageal varix | tracheostomy | negative | coronary artery |
| hepatic encephalopathy | hd | likely | sig |
| rifaximin | solution sig | culture | artery |
| bleed | vancomycin | ed | delay release |
| esophageal | intravenous | cxr | tablet delay |
| octreotide | injection | micu | pound |
| portal vein | dka | iv | particlecrystal |
| hepatitis | abscess | per | sustrel particlecrystal |
| variceal | sig | respiratory | sustrel |

# Appendix 3 – Medical Abbreviations

| **aicd** | automatic implantable cardiac defibrillator |
| --- | --- |
| **alk** | alkaline |
| **am** | am blood tests |
| **ama** | American Medical Association; antimitochondrial antibodies; against medical advice |
| **asd** | atrial septal defect; autism spectrum disorders |
| **ast** | aspartate aminotransferase (formerly SGOT); aspartate transaminase, astigmatism |
| **bid** | twice a day (bis in die) |
| **bipap** | bilevel positive airway pressure |
| **bp** | base pair; blood pressure; bullous pemphigoid |
| **ccu** | critical care unit; coronary/cardiac care unit |
| **cn** | tomorrow night (cras nocte), child nutrition; cognitively normal; cranial nerve |
| **cpap** | continuous positive airway pressure |
| **ct** | chest tube; clinical trial; cognitive therapy; computed/computerized tomography |
| **disp** | dispense |
| **dka** | diabetic ketoacidosis |
| **dm** | dermatomyositis; dextromethorphan; diabetes mellitus |
| **dnr** | do not resuscitate |
| **dvt** | deep venous thrombosis |
| **dz** | disease |
| **ed** | emergency department; erectile dysfunction; effective dose |
| **esrd** | end-stage renal disease |
| **gi** | gastrointestinal; glycemic index |
| **hct** | hematocrit |
| **hd** | health department; hearing distance; hemodialysis; herniated disk; Hodgkin's disease; Huntington's disease |
| **hr** | hour, heart rate |
| **icd** | International Classification of Diseases; implantable cardioverter defibrillator; intrauterine contraceptive device; ischemic cardiac disease |
| **inr** | international normalized ratio |
| **iv** | intravenous |
| **lb** | live birth / lower body / pound |
| **lb** | pound |
| **le** | left eye; lower extremity; lupus erythematosus |
| **mcg** | microgram |
| **min** | minute |
| **mmhg** | millimeter of mercury |
| **mr** | medical record / mental retardation / mitral regurgitation / magnetic resonance |
| **neg** | negative |
| **nh** | nursing home |
| **pcp** | primary care physician; pneumocystis carinii pneumonia; phencyclidine (anesthetic/hallucinogenic); primary care provider |
| **peg** | percutaneous endoscopic gastrostomy; pneumoencephalogram; polyethylene glycol |
| **ph** | public health; parathyroid hormone; past history; poor health, hydrogen ion concentration (measure of acidity/alkalinity) |
| **picc** | peripherally inserted central catheter |
| **po** | by mouth / per os |
| **ppi** | proton pump inhibitor: patient package insert |
| **ptt** | partial thromboplastin time |
| **qd** | every day (quaque die), once daily |
| **qid** | 4 times a day |
| **rdw** | red cell distribution width |
| **rle** | right lower extremity |
| **recon soln** | solution reconstituted |
| **rr** | recovery room; relative risk; respiratory rate |
| **sig** | “signetur” (let it be labeled) surface immunoglobulin / write on label |
| **staph** | staphylococcal infections |
| **stent** | Subclass of:Prostheses and Implants |
| **th** | thyroid hormone |
| **tid** | 3 times a day / ter in die |
| **trach** | tracheotomy; trachea(l); tracheostomy |

# Appendix 4 – LOS Distribution


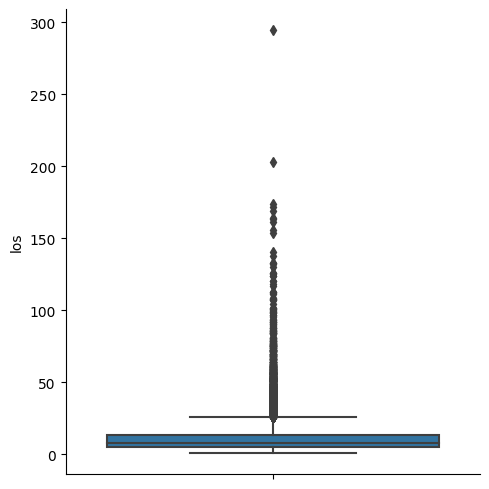

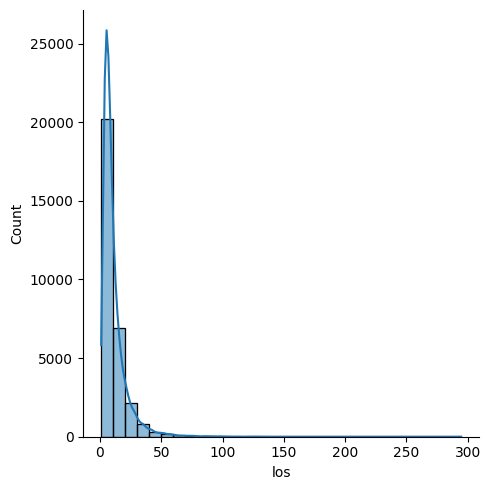

Supplement: S1 File — (DOCX) [file pone.0289795.s001.docx]
