# Supplementary material for: Finding the best trade-off between performance and interpretability in predicting hospital length of stay using structured and unstructured data
Source: PLoS One. 2023 Nov 30;18(11):e0289795. doi: 10.1371/journal.pone.0289795 (PMC10688642; doi:10.1371/journal.pone.0289795)
Supplement: S2 File — (DOCX) [file pone.0289795.s002.docx]

# Supplementary Material

# On the use of absolute LIME weights, and their advantage over their raw values

Displaying the top positive and top negative feature is a common practice when using LIME for local explanation, i.e., for individual instances (1,2). However, when extending it to many instances, to get information on global (variable) explanation, the practice is to average the absolute value of the weights on an appropriate subset of the full dataset ⸺as illustrated in the Submodular Pick approach (3)⸺ or on all the dataset as we have done in this paper.

Why use the absolute value of the weights rather than the raw values when moving from local to global explanation?

Let us use an analogy. When measuring the overall error of a linear model using Ordinary Least Square, the overall error of prediction is measured either by the mean absolute error or the mean square error (the error being the difference between the actual and predicted values of the outcome). Why not just take the aggregated average raw errors? Because the negative errors and the positive errors will cancel each other. The same issue arises for the coefficients: for some instances the same token will have a positive coefficient, and for others it will have negative coefficients. The averaging will thus erase the information contained in the data. The proof for this is in the following Figure 1.

| 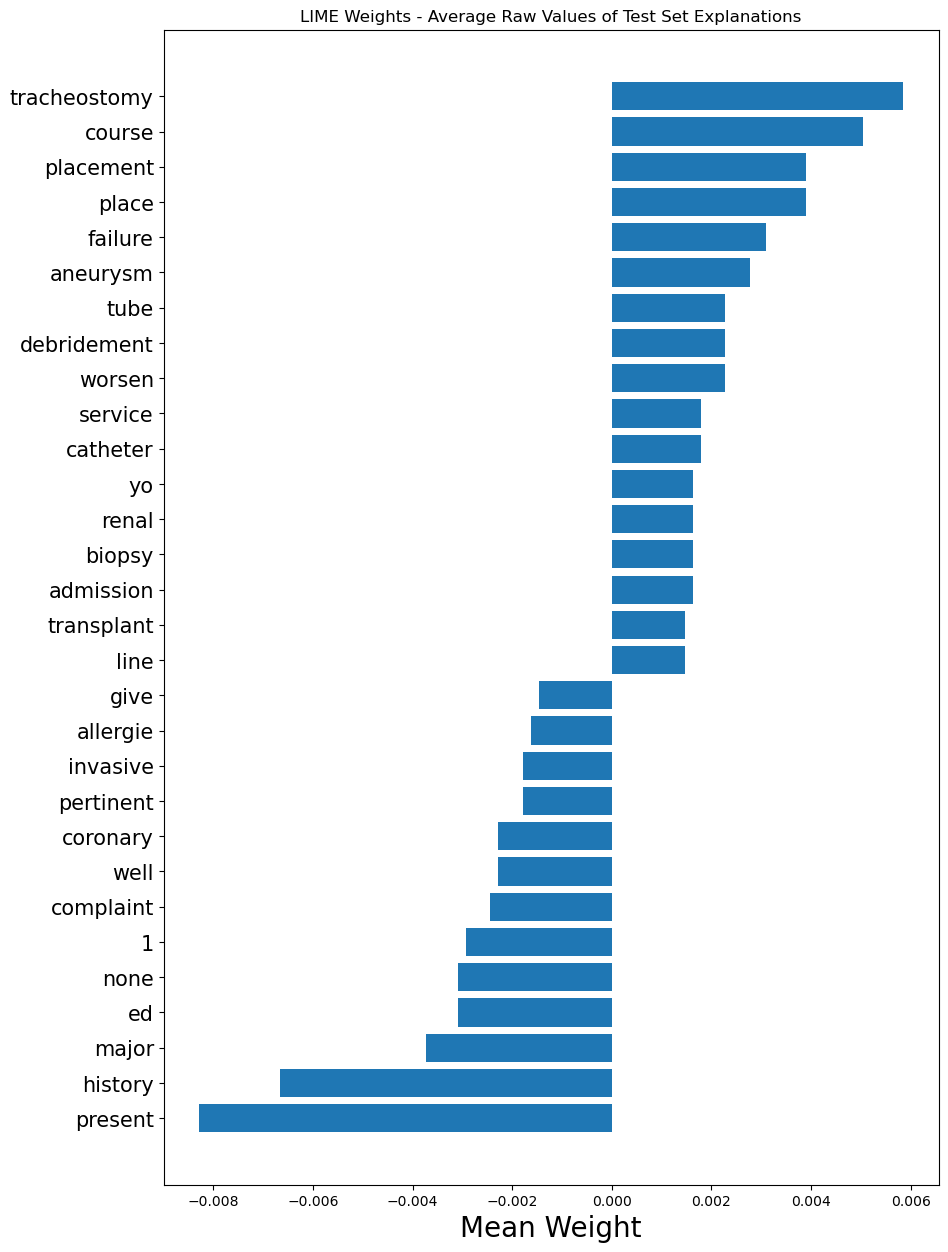 | 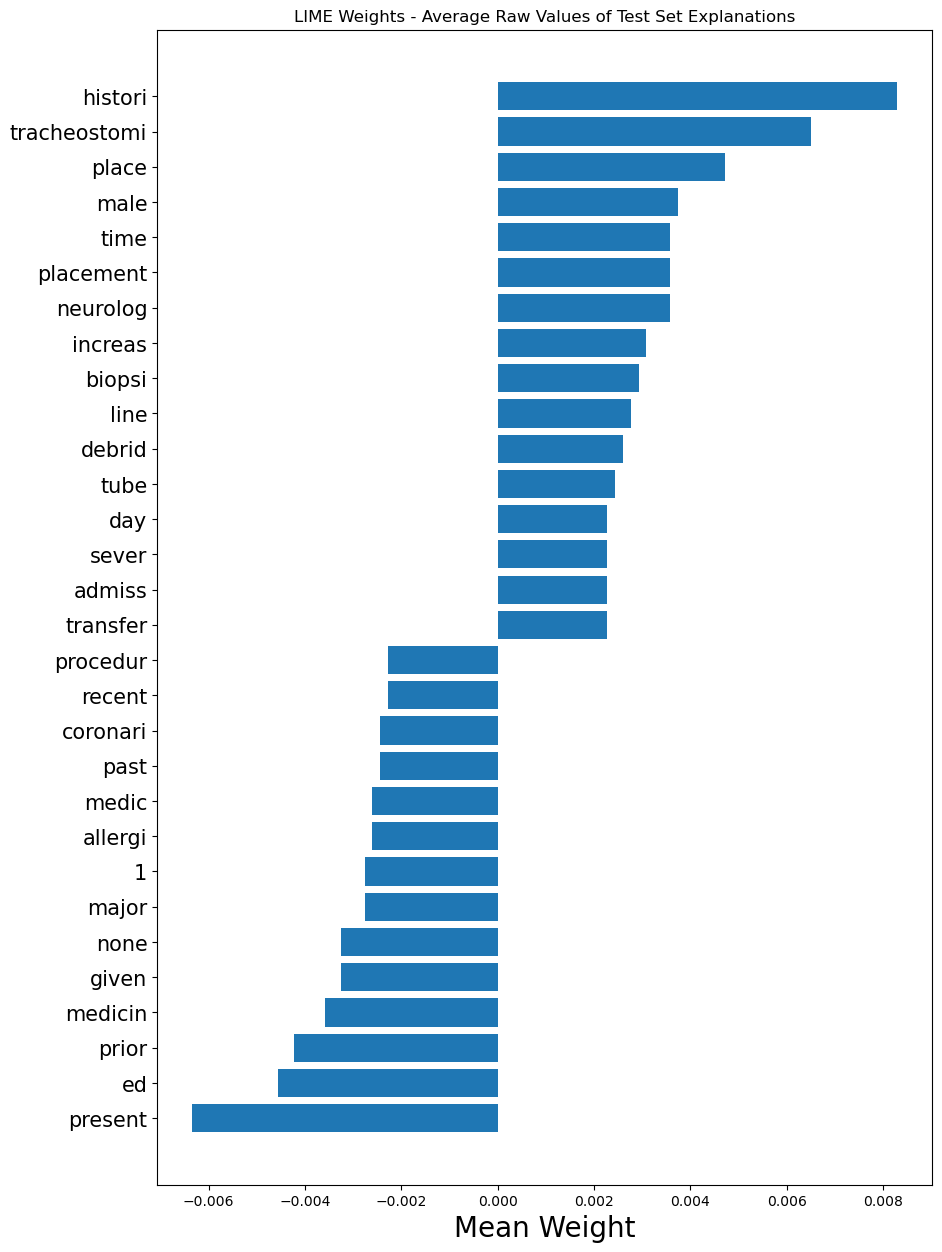 |
| --- | --- |

Figure 1- Averaged local (LIME) raw values feature importance for the BERT Transformer

When we examine the token <history>, on the lemmatized tokens it is a mitigating factor for PLOS (negative coefficient); whereas <history> is an aggravating factor for PLOS. Of course, this sign difference in coefficients may be explained by the difference in covariate tokens (and intercepts) between the two preprocessing approaches, however, when examining the two tokens in context, they are very comparable (see Table 1).

The authors feel that including the averaged raw weights values can be not only confusing but also misleading for the readers, for this reason, in the main paper, we provided only the absolute weights.

| **History (lemma)** | | | | **Histori (stemming)** | | | |  |
| --- | --- | --- | --- | --- | --- | --- | --- | --- |
| **Preceding Words** | **freq** | **Following Words** | **freq** | **Preceding Words** | **freq** | **Following Words** | **freq** | |
| past | 8793 | illness | 6564 | past | 8935 | ill | 6623 | |
| history | 3891 | history | 3912 | histori | 4278 | histori | 4298 | |
| procedure | 2017 | physical | 3233 | procedur | 2262 | physic | 3578 | |
| year | 1915 | exam | 2923 | year | 2006 | exam | 3214 | |
| medical | 1719 | year | 2764 | medic | 2005 | year | 2853 | |
| social | 1611 | sp | 1930 | invas | 1852 | sp | 2115 | |
| family | 1574 | yo | 1757 | surgic | 1747 | live | 2069 | |
| use | 1513 | patient | 1749 | famili | 1712 | present | 1906 | |
| drug | 1490 | disease | 1643 | social | 1709 | patient | 1876 | |
| invasive | 1466 | old | 1502 | deni | 1601 | diseas | 1826 | |
| deny | 1411 | 2 | 1472 | ill | 1595 | yo | 1772 | |
| surgical | 1386 | present | 1455 | use | 1561 | old | 1741 | |
| patient | 1363 | family | 1418 | patient | 1557 | 2 | 1605 | |
| illness | 1340 | etoh | 1112 | drug | 1524 | famili | 1552 | |
| etoh | 1275 | live | 1099 | present | 1472 | male | 1352 | |
| present | 1134 | male | 1060 | etoh | 1323 | etoh | 1175 | |
| sp | 1040 | artery | 1029 | major | 1210 | arteri | 1074 | |
| old | 963 | die | 1007 | sp | 1157 | hypertens | 1069 | |
| none | 955 | use | 970 | old | 982 | htn | 1057 | |
| tobacco | 837 | htn | 947 | none | 970 | cad | 1052 | |

Table 1 – Comparing <history> (lemmatized) and <histori> (stemmed) tokens in context

# On generalizability, age and LOS

In our main paper, in Table 1, older age seems to be associated with shorter LOS (RLOS). This result may appear counterintuitive when comparing our study with similar research done in European countries, such as in a recent study conducted in a University Hospital in the South of France (4) where older patients experience longer LOS. Our data are essentially based on ICU patients from the Boston Beth Israel Deaconess Center, which is a very different type of institution with respect to comparable European studies.

To investigate the origin of this seemingly discrepancy, we conducted a series of chi-square tests between several of the categorical variables present in our dataset and report them in Table 2.

We note that:

1- There are indeed significantly fewer PLOS admissions for the 65-84 and 85+ years categories than would be otherwise expected under hypothesis of independence.

2- There are significantly more Emergency admissions for the 18-44 and 85+ years categories, but significantly less Emergency admissions in the 45-64 and 65-84 years categories than expected.

3- There are significantly more Elective (planned) admissions for the 45-64 and 65-84 years categories, but significantly less Elective admissions in the 18-44 and 85+ years categories than expected

4- There are significantly more PLOS admissions in the Emergency and Urgent admissions, and significantly less Elective (Planned) admissions than expected.

All the aforementioned results point out how, in our data, most PLOS are due to Emergency or Urgent admissions and concern the 18-44 years and 85+ years, which are smaller in proportion.

The 45-64 years and 65-84 years categories are mostly in the planned admissions, thus more likely to be in the RLOS category, and larger in proportion.

As mentioned in the paper Discussion section, our results may be specific to the hospital we chose to study and thus may not be easily generalizable, and this analysis further proves this limitation of our study. However, as discussed in the same section, our main goal is to provide a methodology and a process (i.e., combining structured and unstructured data and utilizing XAI methods to provide a better understanding of the predictions) that can be easily transferred and adapted to a different setting.

| **Age \ LOS** | **RLOS** | **PLOS** |
| --- | --- | --- |
| 18-44 years | 3629  (3715) [-1.417] | 378  (291) [5.058] |
| 45-64 years | 9573  (9749) [-1.790] | 942  (765) [6.388] |
| 65-84 years | 12186  (12053) [1.204] | 814  (946) [-4.296] |
| 85+ years | 3137  (3006) [2.388] | 105  (235) [-8.525] |
| **Chi Square** | 169.885 |  |
| **p-value** | 0 |  |

| Age \ Admission | ELECTIVE | EMERGENCY | URGENT |
| --- | --- | --- | --- |
| 18-44 years | 394  (636) [-9.596] | 3532  (3305) [3.942] | 81  (65) [1.895] |
| 45-64 years | 1953  (1668) [6.952] | 8394  (8673) [-3.004] | 168(172) [-0.325] |
| 65-84 years | 2311  (2063) [5.450] | 10474  (10723) [-2.41] | 215  (212) [0.139] |
| 85+ years | 225  (514) [-12.766] | 2977  (2674) [5.853] | 40  (53) [-1.799] |
| Chi Square | 404.679 |  |  |
| p-value | 0 |  |  |

| Admission \ LOS | RLOS | PLOS |
| --- | --- | --- |
| ELECTIVE | 4654  (4527) [1.878] | 229  (355) [-6.704] |
| EMERGENCY | 23435  (23530) [-0.620] | 1942  (1846) [2.212] |
| URGENT | 436  (467) [-1.449] | 68  (36) [5.171] |
| Chi Square | 82.590 |  |
| p-value | 0 |  |

Table 2 – Contingency tables for Age, LOS category and Admission type. We display actual values and provide expected frequencies in parenthesis and residuals in square brackets. Moreover, we provide the chi-square and p-value of the bivariate tests.

**References**

1. Mardaoui D, Garreau D. An Analysis of LIME for Text Data. In: Proceedings of The 24th International Conference on Artificial Intelligence and Statistics [Internet]. PMLR; 2021 [cité 26 mai 2023]. p. 3493‑501. Disponible sur: https://proceedings.mlr.press/v130/mardaoui21a.html

2. Visani G, Bagli E, Chesani F, Poluzzi A, Capuzzo D. Statistical stability indices for LIME: obtaining reliable explanations for Machine Learning models. J Oper Res Soc. 2 janv 2022;73(1):91‑101.

3. Ribeiro MT, Singh S, Guestrin C. « Why Should I Trust You? »: Explaining the Predictions of Any Classifier. In: Proceedings of the 22nd ACM SIGKDD International Conference on Knowledge Discovery and Data Mining [Internet]. New York, NY, USA: Association for Computing Machinery; 2016 [cité 24 juin 2023]. p. 1135‑44. (KDD ’16). Disponible sur: https://dl.acm.org/doi/10.1145/2939672.2939778

4. Jaotombo F, Pauly V, Fond G, Orleans V, Auquier P, Ghattas B, et al. Machine-learning prediction for hospital length of stay using a French medico-administrative database. J Mark Access Health Policy. 31 déc 2023;11(1):2149318.
