# Supplementary material for: Finding the best trade-off between performance and interpretability in predicting hospital length of stay using structured and unstructured data
Source: PLoS One. 2023 Nov 30;18(11):e0289795. doi: 10.1371/journal.pone.0289795 (PMC10688642; doi:10.1371/journal.pone.0289795)
Supplement: S3 File — (DOCX) [file pone.0289795.s003.docx]

**Supporting Information**

The codes used for this Research is publicly available a the following github address : <https://github.com/jaotombo/LOS_mixed_2022>

The MIMIC III database is also freely available but requires a special registration, as stated here : <https://mimic.mit.edu/docs/gettingstarted/>
